# Ultrasensitive 3D Stacked Silicon Nanosheet Field-Effect Transistor Biosensor with Overcoming Debye Shielding Effect for Detection of DNA

**DOI:** 10.3390/bios14030144

**Published:** 2024-03-14

**Authors:** Yinglu Li, Shuhua Wei, Enyi Xiong, Jiawei Hu, Xufang Zhang, Yanrong Wang, Jing Zhang, Jiang Yan, Zhaohao Zhang, Huaxiang Yin, Qingzhu Zhang

**Affiliations:** 1 School of Information Science and Technology, North China University of Technology, Beijing 100144, China; liyinglu@ime.ac.cn (Y.L.); mikuvoice01@163.com (E.X.); 13811571392@163.com (J.H.); zhangxufang@ncut.edu.cn (X.Z.); wangyanrong@ncut.edu.cn (Y.W.); zhangj@ncut.edu.cn (J.Z.); yanjiang@ncut.edu.cn (J.Y.); 2 Advanced Integrated Circuits R&D Center, Institute of Microelectronic of the Chinese Academy of Sciences, Beijing 100029, China; yinhuaxiang@ime.ac.cn (H.Y.); zhangqingzhu@ime.ac.cn (Q.Z.)

**Keywords:** 3D stacked device, SiNW, biosensors, Debye shielding effect, ultra-sensitive detection, DNA

## Abstract

Silicon nanowire field effect (SiNW-FET) biosensors have been successfully used in the detection of nucleic acids, proteins and other molecules owing to their advantages of ultra-high sensitivity, high specificity, and label-free and immediate response. However, the presence of the Debye shielding effect in semiconductor devices severely reduces their detection sensitivity. In this paper, a three-dimensional stacked silicon nanosheet FET (3D-SiNS-FET) biosensor was studied for the high-sensitivity detection of nucleic acids. Based on the mainstream Gate-All-Around (GAA) fenestration process, a three-dimensional stacked structure with an 8 nm cavity spacing was designed and prepared, allowing modification of probe molecules within the stacked cavities. Furthermore, the advantage of the three-dimensional space can realize the upper and lower complementary detection, which can overcome the Debye shielding effect and realize high-sensitivity Point of Care Testing (POCT) at high ionic strength. The experimental results show that the minimum detection limit for 12-base DNA (4 nM) at 1 × PBS is less than 10 zM, and at a high concentration of 1 µM DNA, the sensitivity of the 3D-SiNS-FET is approximately 10 times higher than that of the planar devices. This indicates that our device provides distinct advantages for detection, showing promise for future biosensor applications in clinical settings.

## 1. Introduction

The detection of biomolecules, particularly nucleic acids, is of great importance for research into genetic expression [1], pharmacogenomics [2], drug discovery [3] and molecular diagnosis [4,5,6]. Therefore, the detection methods for nucleic acid molecules have been intensively investigated; they include molecular hybridization techniques, polymerase chain reaction, electrical biosensors, etc. [7,8]. Among them, electrical biosensors, especially the field-effect transistor (FET)-based biosensors, are attracting more and more attention. They can act as a label-free and highly sensitive platform for biomolecular sensing. Moreover, due to the good biocompatibility of the FET-based biosensors with integration of the complementary metal-oxide-semiconductor (CMOS), they are very promising for mass production [9,10,11,12,13]. In particular, silicon nanowire field effect (SiNW-FET) biosensors exhibit great potential because of their high surface-to-volume ratio (S/V) and ease of surface modification [14,15,16,17,18,19,20,21,22,23,24]. They enable us to achieve sensing detection by altering the probe molecules on the nanosensing surface and employing the gate pressure change caused by the probe’s attachment to the target molecule [25].

**Figure 1:**
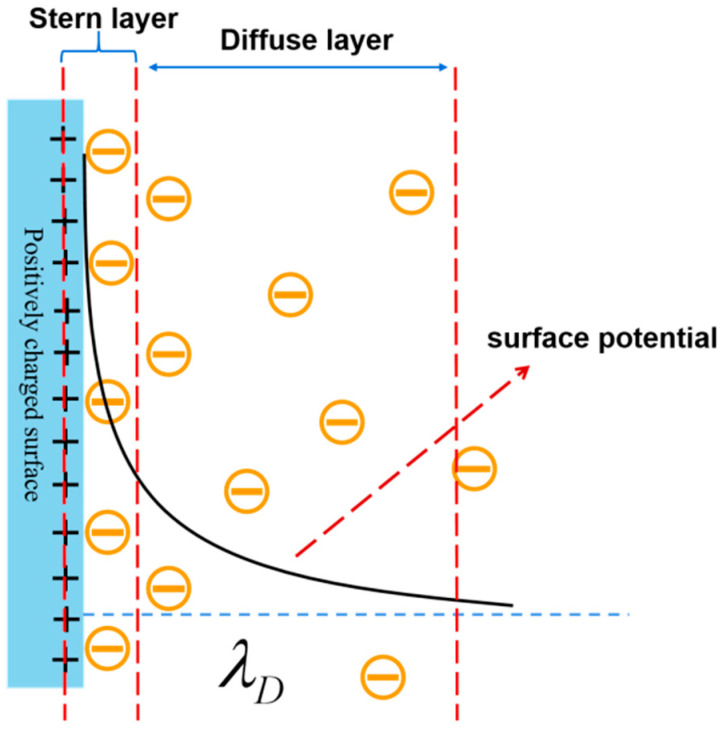
Schematic diagram of the electric double layer (EDL) and the corresponding exponential decline of the surface potential when increasing the distance [38].

However, the Debye shielding of molecular charges by counterions in solution would occur in the FET-based biosensors, which would lower the detection limit [26,27]. Note that an “electric double layer” (EDL) would be formed on the surface of the active probe when detecting biomolecules in a liquid environment. The formation of EDL is caused by the analyte electrically drawing counterions from the ionic solvent, as shown in Figure 1. Additionally, the surface charge of the biomolecule in the buffer solution is surrounded by opposing charges in solution, which causes an exponential decay of the potential with distance. According to the Debye theory, when a field-effect biosensor is used to detect biomolecules, the target molecule’s charge field effect will be shielded by the electric double layer when the distance between the target molecule and the sensing surface exceeds the length of the Debye (thickness of the electric double layer) [28,29]. This means that the target molecule’s charge field effect will not be able to effectively influence the distribution of the carriers in the channel, which will cause a change in the electric current. This phenomenon is known as the Debye shielding effect. Therefore, overcoming Debye shielding is one of the major issues that needs to be addressed for FET-based biosensors [30,31]. So far, some strategies have been proposed to overcome the Debye shielding effect, mainly from three aspects: increasing the Debye length, optimizing the target, and optimizing the device structure. For example, with the desalination and filtration of serum using micro hemodialysis, the dilution of the ionic strength of the solution effectively increases the Debye length, but it will have a certain impact on the activity of the sample [32,33]; the shortening of the distance of the target from the sensing surface occurs by pruning the antibody, but it is more complicated to prepare [34]; there are also the methods that entail replacing the functionalized antibody on the silicon nanowire with an aptamer [35]. Also, the incorporation of biomolecule-permeable polymers into semiconducting materials in order to change the dielectric constant can be effective in overcoming a certain degree of Debye shielding effect [36]. However, with the exception of Hwang et al., who reported the use of pleated graphene to overcome the effect of Debye shielding effect, few studies have been conducted on overcoming the Debye shielding effect from the point of view of optimizing devices [37].

Considering the above problems, in this work, a three-dimensional stacked silicon nanosheet FET (3D-SiNS-FET) biosensor was designed and fabricated with the aim of improving the spatial architecture of the device. The biomolecules are immobilized in the internal cavity of the 3D stacked structure through surface modification, so that the upper and lower sensing surfaces just capture the target biomolecules within the length range of the opposite side of the Debye, achieving complementary detection of the upper and lower sides. Thus, this device architecture can both solve the Debye shielding and realize high-sensitivity detection. Prior to the device’s fabrication, COMSOL simulation was performed to verify its feasibility. The detection results show that the sensitivity of the 3D-SiNS-FET biosensor is approximately 10 times higher than that of the planar device in a highly concentrated 1 µM solution of long-stranded DNA, and the DNA detection limit has reached the lowest level ever recorded at less than 10 zM, demonstrating that the device enables us to not only eliminate the Debye shielding effect, but also to achieve optimal sensitivity detection.

## 2. Materials and Methods

### 2.1. Materials and Reagents

Phosphate buffer solution (PBS), anhydrous ethanol and 1-(3-dimethyl(yl)aminopropyl)-3-ethylcarbodiimide hydrochloride (EDC) were obtained from Sigma-Aldrich (St. Louis, MI, USA), and N-hydroxysuccinimide (NHS) was purchased from Inokai Technology Co. (Tokyo, Japan). The primer DNA was provided by Beijing Biotech (Beijing, China) and designed with a 6-base complementary DNA sequence CAC AAC and a nucleic acid base sequence GTG TTG. The 12-nucleotide primer DNA sequence was produced using the complementary sequence of CAA CCA CAC AAC and the nucleic acid base sequence of GTT GGT GTG TTG. The length of the DNA was 2.04 nm and 4.08 nm for 6 and 12 bases, respectively. In this study, qRT-PCR was used to verify the accuracy of the DNA target sequences to be tested (See Figure S2). To prepare PBS solutions with different pH values, a drop of PBS solution was added gradually to PBS solutions with various pH levels, which were then monitored in real-time with a pH meter. The pH configuration was established using HCl and NaOH.

### 2.2. Design and Fabrication

**Figure 2:**
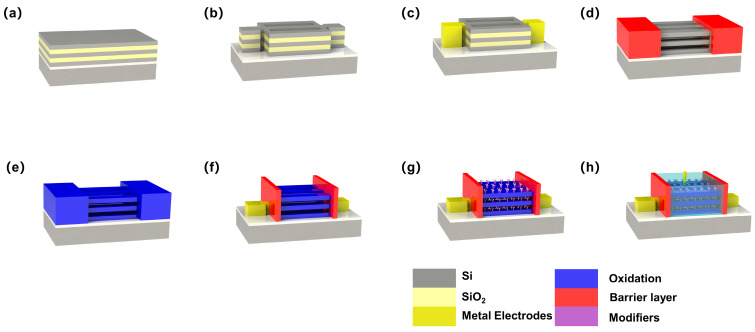
Main fabrication flow of a 3D-SiNS-FET biosensor: (**a**) SiO_2_/Si stack growth, (**b**) SiO_2_/Si stack structure patterning, (**c**) Source–drain metallization, (**d**) SiO_2_ stack removal, (**e**) Gate oxide growth, (**f**) Source–drain electrode, (**g**) 3D-NS-FET specific modification, and (**h**) 3D-SiNS-FET device scheme.

The 3D-SiNS-FET biosensor was fabricated and designed utilizing the CMOS platform. The process starts by epitaxial etching of the silicon wafer surface to choose the higher two materials, followed by the formation of the desired SiNW and SiNS structure using photolithography patterning techniques. Subsequently, one of the epitaxial materials was removed using selective etching techniques. Finally, the gate medium was formed and filled with metal using atomic layer deposition (ALD). Figure 2 displays the significant stages in the preparation of a 3D-SiNS-FET biosensor.

The silicon-on-insulator (SOI) structure was first fabricated on a silicon (100) substrate by thermal deposition of a 145 nm SiO_2_ BOX layer and a 20 nm α-Si top layer. Next, to construct a three-dimensional stacked structure utilizing Si and SiO_2_ as the bonded layers, 20 nm-thick α-Si and 40 nm-thick SiO_2_ stacked layers were deposited on the SiO_2_ surface by plasma-enhanced chemical vapor deposition (PECVD). The fundamental Si nanosheet structure was created by dry etching under strict supervision and standard photolithography (see Figure 2b). To reduce the contact resistance of the source–drain area, a nickel–platinum alloy (refer to Figure 2c) was applied onto the surface to generate a metal silicide. The most important process of the device preparation process was the removal of the SiO_2_ stack. To ensure that the source–drain areas would not be etched during the SiO_2_ stack etching, SiO_2_ and SiN_x_ layers were first deposited onto the surface as a mask to protect the source–drain electrodes (see Figure 2d). In the following, the channel section was exposed through step-by-step etching of SiO_2_ and SiN_x_ layers, and selective wet etching with a solution of HF (6%):CH_3_COOH (99.8%):H_2_O_2_ (30%) with a ratio of 1:3:2. Next, 3D-SiNS-FET biosensors were prepared with an optimal pitch (Figure 2d). To fulfill the requirements of liquid environment detection, ALD was employed to fabricate a high-k hafnium oxide (HfO_2_) layer as the dielectric to prevent leakage during liquid detection.

### 2.3. Surface Modification

**Figure 3:**
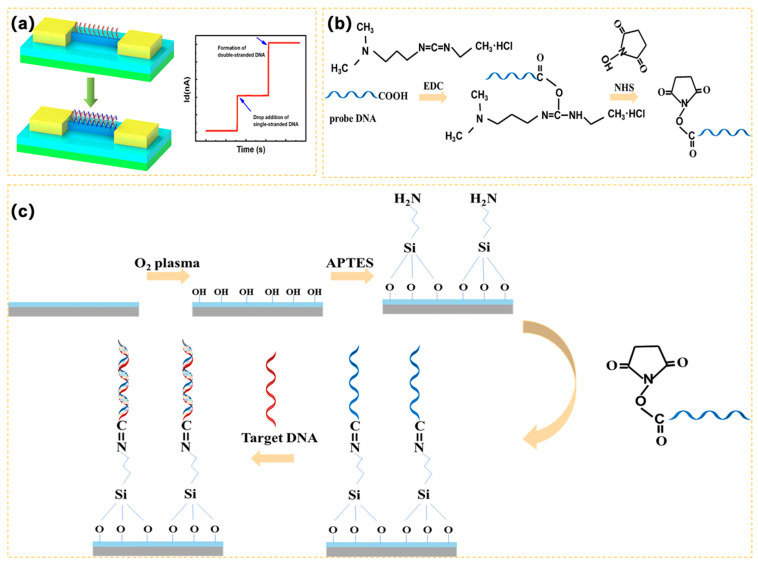
(**a**) Detection principle of the 3D-SiNS-FET biosensor; (**b**) Schematic diagram of EDC/NHS activation of carboxyl groups; (**c**) Surface modification and detection process of 3D-SiNS-FET biosensor.

To maintain the sensitivity and specificity of biosensors, the surface modification is critical. Accordingly, a single-strand DNA was employed to achieve firmly covalent binding with the surface of 3D-SiNS-FET. Specifically, the sensor surface was treated with oxygen plasma for 3 min to create more hydrophilic surface hydroxyl groups after the device surface was previously cleaned with anhydrous ethanol. The device surface was coated with 1% 3-aminopropyltriethoxysilane (APTES) and left for 30 min to allow the hydroxyl groups on the surface to bond with the siloxyl groups in the APTES. The unreacted APTES was removed by immersing in anhydrous ethanol. The surface was blown with nitrogen and then placed in a vacuum drying oven at 110 °C for 30 min to cure the silylation. At room temperature, the single-stranded DNA was combined with EDC and NHS solution in a 2:1 molar ratio. The combination was then spread over the surface of the device and allowed to sit for 1 h. As a result of the reaction of NHS and EDC, the terminal carboxyl group (-COOH) of the DNA probe was activated (see Figure 3b), which facilitated condensation interaction with the amino group on the sensor surface. It was then dried with nitrogen for storage followed by cleaning with buffer and DI water.

### 2.4. Electrical Detection of DNA

The specificity, sensitivity and stability of the 3D-SiNS-FET biosensor were electrically characterized by using a current-voltage (I–V) measurement system (Agilent B1500A, Keysight, Santa Rosa, CA, USA). An S4800 scanning electron microscope (SEM) was used to confirm the morphological quality of 3D-SiNS-FETs. For real-time monitoring of target DNA at different concentrations and lengths in the range of 1 zM to 1 µM, *V*_d_ = −5 V and *V*_g_ = −10 V were applied to the 3D-SiNS-FET biosensor. To start the detection process, the sensor’s surface was treated with 1.5 µL of PBS buffer to determine the baseline current I_0_. The corresponding currents of different DNA concentrations, *I*_i_, were then established by adding the same volume of a specific concentration of DNA solution and gradually increasing the concentration from low to high. The rate of change of currents of different DNA concentrations was then computed by calculating (*I*_i_ − *I*_0_)/*I*_0_. A large number of carriers (holes) accumulate within the SiNS when more negatively charged DNA single strands are captured by the probes on the surface of the SiNS in the p-type device (similar to the application of a positive gate voltage), which causes an increase in the *I*_SD_ (see the working schematic in Figure 3a).

## 3. Results and Discussion

### 3.1. Simulation

**Figure 4:**
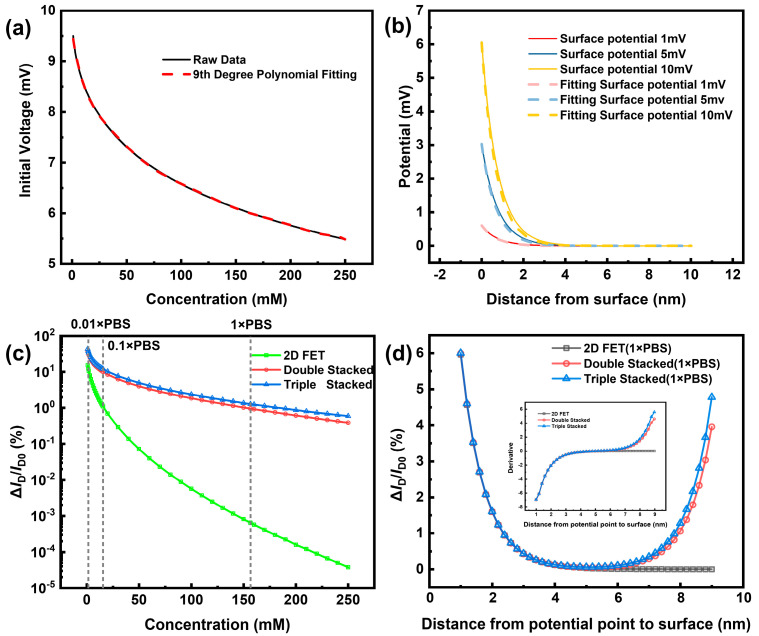
The results of the COMSOL simulation. (**a**) Surface potential remaining after lowering by a narrow EDL potential at different concentrations. (**b**) Decreasing trend and curve fitting of a diffusion EDL potential under different surface potentials. (**c**) Simulation of 2D NW sensitivity at different concentrations compared to double- and triple-stacked NW (**d**) Simulation of 2D NW sensitivity compared to double- and triple-stacked NW using constant DNA strand length to vary NW spacing.

In prior to the 3D-SiNS-FET fabrication, the simulation of the 3D stacked structure was performed using COMSOL Multiphysics software to confirm whether the Debye shielding effect can be eliminated. In the simulation, the EDL formation and corresponding Debye shielding effect were considered. Using coupled electrostatic field and dilute matter diffusion field models, two ions, Na^+^ and Cl^−^, are defined, and their concentrations are varied to simulate solution environments with different concentrations. Figure 4a displays the residual potential resulting from the passage of a 10 mV surface potential through the tight EDL at varying ionic strengths. It is clear from the figure that the tight EDL has a greater effect on the potential landing in the high ion concentration environment, and the tight EDL rapidly reduces the surface potential to 1/e of the initial potential to the limit of the Debye length. The surface potentials defined in Figure 4b all drop to almost zero after a distance of more than 2 nm. This suggests that in biomolecule detection, most of the charge to be detected is shielded, resulting in a very small current change due to the Debye shielding effect.

To determine whether stacked-structured nanowires had an advantage in sensitivity, 2D NW, double-stacked NW, and triple-stacked NW were first simulated for sensitivity at varied concentrations. The sensitivities of both 2D NW and both types of stacked NW gradually decline with increasing concentration, as shown in Figure 4c, and the difference in sensitivity between 2D NW and stacked NW grows larger and larger with increasing concentration. When compared to 2D NW, Triple-Stacked NW showed a 3-fold increase in sensitivity from 0.01 × PBS, an 11-fold increase in sensitivity from 0.1 × PBS, a 1990-fold increase in sensitivity at 1 × PBS, and a massive 15,815-fold increase in sensitivity at 250 mM. Second, simulation was used to investigate the sensitivity of DNA with varying strand lengths at a fixed 10 nm spacing. As can be seen in Figure 4d, the stacked NW exhibits a very different trend from the 2D NW with the continuous increase in chain length after 5 nm from the sensing surface. Specifically, the stacked NW’s sensitivity begins to rebound, while the 2D NW’s sensitivity keeps decreasing, and after 6 nm, the 2D NW’s sensitivity is almost zero (the rate of change of sensitivity is shown in the figure’s inset). This indicates the spatial advantage of the top and bottom complementary detection of its three-dimensional stacked structure.

The simulation results confirm that at extremely low to very high concentrations, stacked NW clearly outperforms 2D NW, and that the advantage of stacked NW increases with concentration. Furthermore, it is evident that stacked NW has a markedly higher rate of change for the long-chain chemicals that need to be measured.

### 3.2. Structural Characterization and Electrical Characteristics of 3D-SiNS-FET Sensor

**Figure 5:**
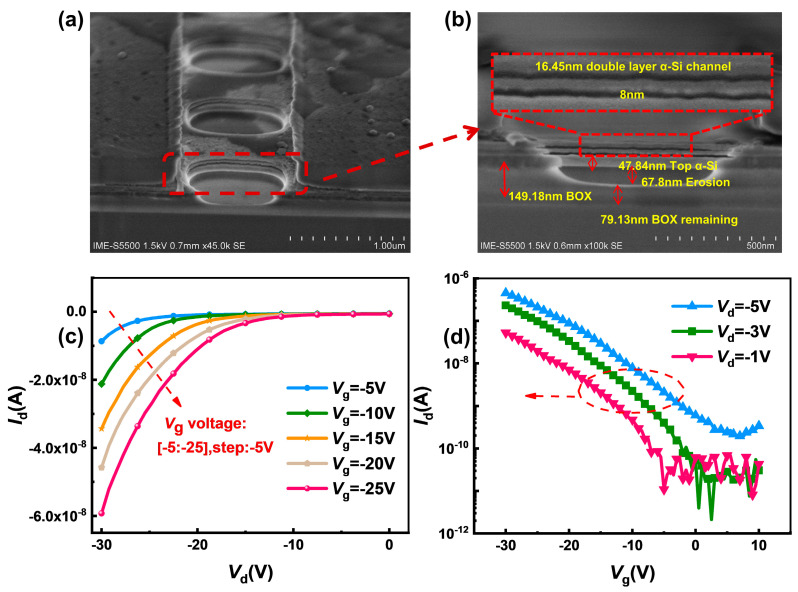
Structural features and electrical characteristics of the device. (**a**,**b**) show a 30-degree top view of the trench portion and a partial enlargement of the trench portion of the 3D-SiNS-FET biosensor, respectively, and (**c**,**d**) show the output characteristics at different gate pressures as well as the transmission characteristics at different gate pressures, respectively.

To optimize stack release and preparation efficiency, the Si/SiO_2_ structure was used to prepare the stack, and HF acted as the etchant due to its ultra-high selectivity ratio of SiO_2_ over Si. Figure 5a shows a typical SEM image of our 3D-SiNS-FET sensor with a channel length of 1 µM and a width of 500 nm, and it is clear that the spatial 3D stacked structure was successfully formed. To show the details and specific parameters of the structure, the front-view SEM image was partially enlarged, as illustrated in Figure 3b. After HF etching, the channel width was shrunk to 8 nm, leaving a double Si-channel layer with thickness of 16.45 nm and a 79.13 nm-thick BOX layer to prevent the physical contact between the substrate and the solution dropped.

Using the Keysight B1500, the basic electrical characteristics of the 3D SiNS FET biosensor were measured. The output characteristics at different gate voltages are shown in Figure 3c. the device has good output characteristics and is sensitive to changes in gate voltage. The gate voltage (*V*_g_) was scanned from −20 V to −5 V at 5 V intervals, and the drain current (*I*_d_) increases as the drain voltage (*V*_d_) increases for each device. The drain current increases significantly as the negative gate voltage *V*_g_ goes from 0 to −10 V, demonstrating that the 3D-SiNS-FET biosensor has good PMOS characteristics. Figure 5d shows the transfer characteristics at different drain voltages of −1 V, −3 V and −5 V, respectively. The output and transfer characteristics show that the 3D-SiNS-FET biosensor has good gate control capability and is suitable for biosensing testing.

### 3.3. Detection and Sensitivity of 3D-SiNS-FET

**Figure 6:**
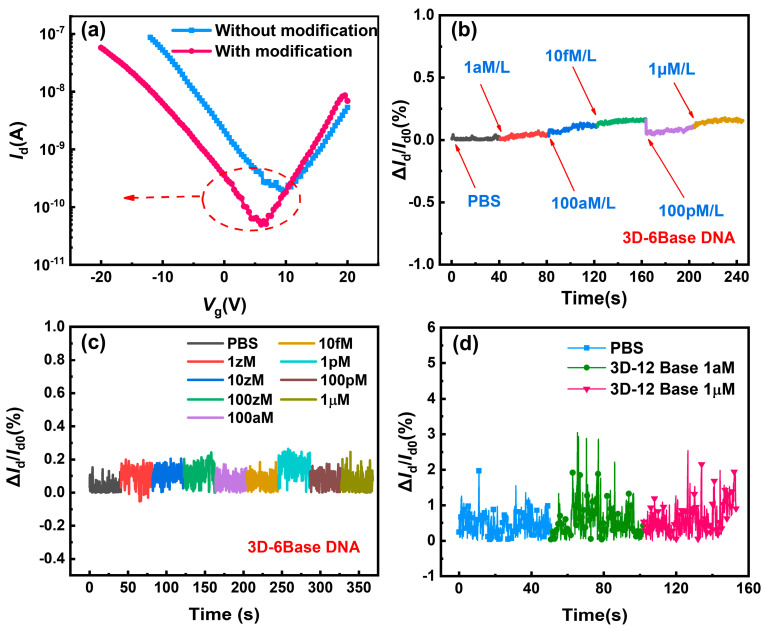
Characterization of modification effects in 3D-SiNS-FET biosensors. (**a**) Transfer characteristic curves of the modified and unmodified devices. (**b**,**c**) Detection of 6-base DNA by the unmodified device. (**d**) Detection of 12-base DNA by the unmodified device.

Before the formal detection of DNA molecules using the devices, the electrical characterization of the same device was conducted before and after modification. As shown in Figure 6a, the threshold voltage shifted left after modification. This would be attributed to the electronegative DNA probe molecule fixed on the device surface, which generated a negative electric field on the gate and thus increased the current in the SiNS channel. This indicates the successful execution of the modification process. Regarding the control group of the unmodified devices, their currents have no significant changes when the concentration of DNA solutions varies, as shown in Figure 6b–d, which is due to the absence of the DNA probe. Thus, we can deduce that the 3D-SiNS-FET biosensor after modification can perform nucleic acid-specific detection.

**Figure 7:**
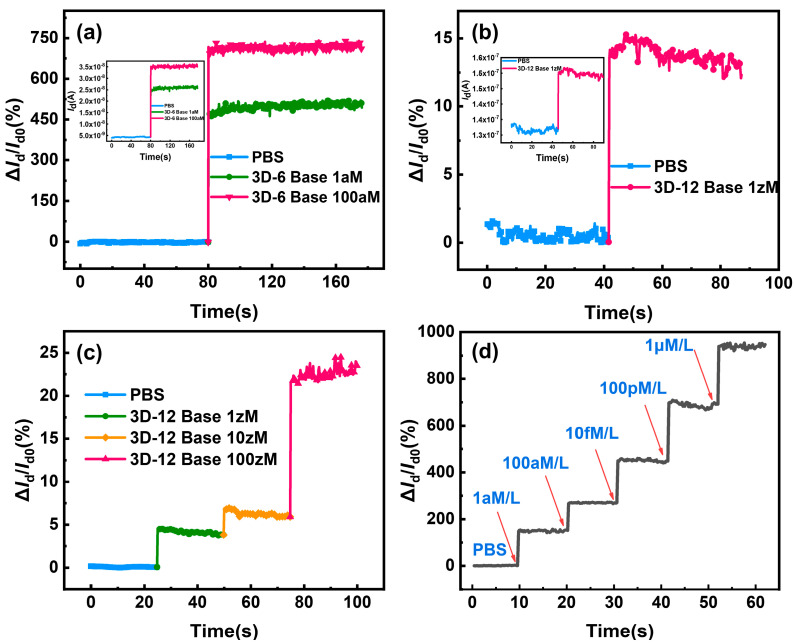
Current rate of change curve and the I-V characteristics for a 3D-SiNS-FET biosensor under the conditions *V*_d_ = −5 V and *V*_g_ = −10 V. Due to the remarkable sensitivity of the surface probe to variations in target DNA concentration, the current rate of change consistently increases across concentrations ranging from 1 aM to 1 µM of the target DNA. (**a**) Current change rate of the 3D-SiNS-FET biosensor in response to 1 aM vs. 100 aM 6-base DNA, with the inset depicting the corresponding real-time current profile. (**b**) Current change rate of the 3D-SiNS-FET biosensor for 1 zM 12-base DNA, accompanied by the inset illustrating the corresponding real-time current dynamics. (**c**) Current change rate of the 3D-SiNS-FET biosensor for a series of concentrations (1 zM, 10 zM, 100 zM) of 12-base DNA. (**d**) Real-time response of the 3D-SiNS-FET biosensor to the rate of change of current for different concentrations of DNA (ranging from 1 aM to 1 µM), with red arrows indicating the injection sites.

The modified device can be employed for hybridization by selectively capturing the target molecules for the sensing detection of various DNA concentrations once it has been confirmed that the sensing surface of the device has been successfully transformed with DNA probe molecules. Figure 7a illustrates the remarkable current changes and high current change rate of approximately 450% and 700%, respectively, upon hybridization of 1 aM and 100 aM of complementing six-base DNA with probe-functionalized 3D-SiNS-FET. Figure 7d presents the real-time response to different concentrations of 12-base DNA strands. The 3D-SiNS-FET demonstrates a pronounced dose-dependent relationship as the concentration of 12-base DNA strands varies from 1 aM to 1 µM. The Id/Id0 ratio measures approximately 150%, 270%, 450%, 680%, and 930% for DNA solutions at concentrations of 1 aM, 100 aM, 10 fM, 100 pM, and 1 µM, respectively. In accordance with the theoretical framework underpinning the P-type 3D-SiNS-FET biosensor, the observed increase in current signifies that the specific binding of DNA strands results in an equivalent negative charge being introduced to the surface of the SiNS FET biosensor. Furthermore, the calibrated and fitted linear regression equations for six-base DNA and twelve-base DNA can be found, respectively, as y = 686.81229x + 9.48917 with R^2^ = 0.9935 and y = 67.57515x + 756.01819 with R^2^ = 0.99171, where x is the logarithm of DNA concentration. These results demonstrate a strong linear correlation between the rate of change of current and DNA concentration (See Figure S3). And, as can be seen from the detection results in Figure 7b, the three-dimensional stacking device has a 1 zM concentration of 12-base DNA molecules and clearly exhibits a current change rate of about 15% or so, which is the lowest detection limit known for DNA detection (Table 1). Furthermore, at concentrations below 1 aM, the current rate of change displays a noteworthy gradient increase (refer to Figure 7c). The validity of our experimental findings was confirmed when we simultaneously computed the LOD using the three-sigma criteria, LOD (6-Base DNA) ≈ 0.0078 aM = 7.8 zM and LOD (12-base DNA) ≈ 0.00091 aM = 0.91 zM.

The results show that this stacking structure enables the target DNA molecules specifically captured by the modified DNA probe in the stacking gap to be located all within the Debey length range of the contralateral sensing surface so that the Debey shielding effect is greatly weakened, which improves the sensitivity of the bio-detection and realizes the high sensitivity detection at very low concentration. Furthermore, a notable increase in the quantity of changed DNA probes on the sensing surface is made possible by the three-dimensional stacking structure, which raises the target molecule capture rate and, in turn, the current change rate of DNA detection. The suggested three-dimensional stacked structure has many benefits that make it useful for both scientific study and innovative technology.

To further evaluate the advantage of 3D-SiNS-FET biosensors compared with conventional 2D FET biosensors in detecting biomolecules, multiple concentration solutions of DNA with various sizes were measured in this study.

**Figure 8:**
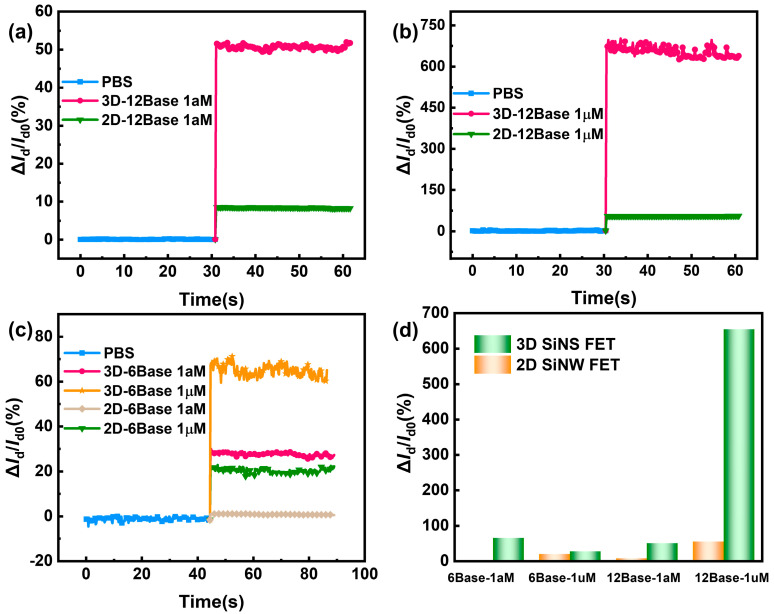
Comparison of 3D and 2D device current rates of change. (**a**,**b**) Comparison of the current change rate of 3D and 2D devices for 12-base DNA at 1 aM and 1 µM concentrations, respectively. (**c**) Comparison of current change rates for 3-D and 2-D devices for 6-base DNA at 1 aM and 1 µM concentrations. (**d**) Summary of comparative assay results.

Firstly, the detection of a high concentration (1 µM) of the 12-base DNA molecule solution demonstrates that the current change rate of the 3D-SiNS-FET biosensor is significantly higher than that of the 2D-FET biosensor, reaching 670% (see Figure 8a). It should be highlighted that at a low concentration (1 aM) of 12-base DNA molecule solution, the 3D-SiNS-FET still shows a significant current variability of about 53%, while the current variability is less than 10% in the 2D FET biosensor (see Figure 8b), suggesting a great clinical application potential of 3D-SiNS-FET due to very rare DNA molecules in the blood stream. Similarly, 3D-SiNS-FET biosensors show higher sensitivity to 6-base DNA molecule solutions, regardless of high (1 µM) or low (1 aM) concentrations (Figure 8c).

**Figure 9:**
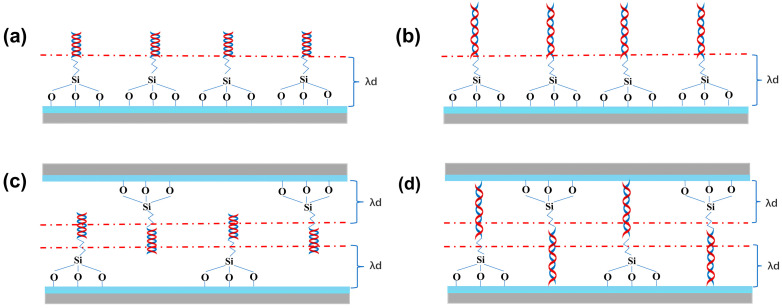
Schematic diagram of DNA detection. (**a**,**b**) Two-dimensional 6-base and 12-base DNA detection device. (**c**,**d**) Schematic of the three-dimensional 6-base and 12-base DNA detection device.

Taken together, compared with 2D-FET biosensors, 3D-SiNS-FET biosensors are clearly more advantageous in detecting 12-base DNA molecules rather than 6-base DNA molecules, with a larger current change rate. As shown in Figure 9a,b and Figure 9c,d, which are the schematic diagrams of the 2D-FET biosensor and 3D-SiNS-FET biosensor, respectively, more DNA strands can enter the Debey length range of 3D-SiNS-FET when detecting 12-base DNA, which alleviates the effect of Debey shielding on its detection and enables the 3D device sensitive to ultra-low concentration samples.

These experimental results highlight the great advantages of 3D-SiNS-FET biosensors for biological detection. In conclusion, the 3D-SiNS-FET biosensor designed and fabricated in this work provides a highly reliable solution for the detection of DNA molecules with different chain lengths and concentrations.

## 4. Conclusions

In conclusion, a 3D-SiNS-FET was proposed and fabricated to overcome the Debye shielding effect in the liquid detection environment by covalently binding the device with DNA of different sizes and concentrations. By evaluating the performance through output current measurement and additional experiments, our device with the 3D stacked structure successfully solved the Debye shielding effect due to the advantage of its 3D spatial structure. Furthermore, the sensitivity of the 3D stacked device was significantly improved compared with the standard 2D device. Furthermore, the sensitivity of the 3D stacked device was significantly improved compared with the standard 2D device. Importantly, this device efficiently detects samples with concentration gradients ranging from 1 nM to 1 µM and reveals the lowest detection limit (below 10 zM) for 12-base DNA (4 nM) in 1 × PBS. These studies promote the potential application of FET biosensors in future clinical diagnostics.

## Figures and Tables

**Table 1:** Biosensors for DNA detection.

Analytical Methods	Material	Detectives	Limit of Detection (LOD)	Reference
Electrode Chemiluminescence (ECL)	DNA- Au@MNPs	DNA	(2 aM to 20 nM) 2 aM	[39]
Electrode Chemiluminescence (ECL)	SiNW array FET	ctDNA	(0.1 fM–100 pM) 10 aM	[40]
Differential Pulse Voltammetry (DPV)	Au@Fe_3_O_4_	circulating tumor DNA	(1 fM–1 nM) 22 aM	[41]
Electrode Chemiluminescence (ECL)	Deformed Graphene	DNA/PNA	600 zM	[37]
Electrode Chemiluminescence (ECL)	3D-SINS-FET	DNA	10 zM	This work

## Data Availability

Data are contained within the article.

## Supplementary Materials

The following supporting information can be downloaded at: https://www.mdpi.com/article/10.3390/bios14030144/s1.
