# Supplementary material for: Ultrasensitive 3D Stacked Silicon Nanosheet Field-Effect Transistor Biosensor with Overcoming Debye Shielding Effect for Detection of DNA
Source: Biosensors (Basel). 2024 Mar 14;14(3):144. doi: 10.3390/bios14030144 (PMC10968264; doi:10.3390/bios14030144)
Supplement: Supplementary file 1 [file biosensors-14-00144-s001.zip › biosensors-2852131-supplementary.pdf]

Supporting information:

1. Simulation

The present work potential simulation is based on the calculation of Poisson equation (Eq. (1-1)).

$$\nabla^2\Phi = -\frac{\rho}{\varepsilon_0} \tag{1-1}$$

Where ▽ is the Hamiltonian operator, Φ is the potential, *ρ* is the charge density, and ε_0 is the vacuum dielectric constant.

The Stern model considers the thickness of the EDL as the distance from the solid-liquid contact surface when the electrostatic potential in the solution is 1/e of the electrostatic potential at the solid-liquid contact surface (the thickness of the EDL is also known as the Debye length), which can be calculated according to (Eq. (1-2)).

$$\lambda_D = \sqrt{\frac{\varepsilon_0 \varepsilon_r k_B T}{cq^2}} \tag{1-2}$$

where λ_D is the Debye length, ε_0 is the vacuum permittivity, ε_r is the relative permittivity, k_B is the Boltzmann constant, *T* is the absolute temperature, *c* is the ionic concentration of the solution, and q is the unit charge.

1.1 *Semiconductor Device Simulation*

The semiconductor devices need to be simulated and analyzed before the biosensing simulation to ensure that they operate in the sensitive subthreshold region. 2D simulation is used for semiconductor simulation, and the basic settings such as doping and length are defined based on the devices prepared in actual production. Specific parameters are shown in Table S1.

Table S1. Device-defined parameters.

**Parameter/Feature**	**Single Nanowire (2D NW)**	**Double-Stacked Nanowire (Double-Stacked NW)**	**Triple-Stacked Nanowire (Triple-Stacked NW)**
Height (nm)	12	12	12
Length (nm)	500	500	500
Source/Drain Doping Depth (nm)	12	12	12
Junction Depth in x-direction (nm)	1	1	1
Body Doping Concentration (cm^3)	1e17	1e17	1e17
Source/Drain Doping Concentration (cm^3)	1e20	1e20	1e20
Spacing Between SiNWs (nm)	Not applicable	15	15
Applied Drain Voltage (mV)	100	100	100
Applied Gate Voltage (V)	3	3	3

100mV drain voltage and 3V gate voltage, the electron concentration distributions of the three types of SiNWs are shown in Fig S1. The source-drain portion as well as the inverse-type layer of the channel can be clearly seen in the figure.

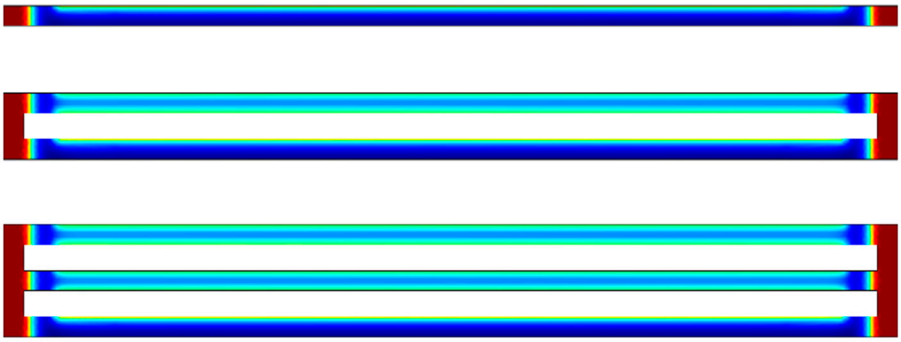

Figure S1. Electron concentration distribution of 2D NW, Double-Stacked NW and Triple-Stacked NW.

1.2 ***The prototype protocol for qRT-PCR is as follows：***

(1) Design of qRT-PCR primers: Based on the 2 DNA sequences to be tested (6-base nucleic acid base sequence GTG TTG. The 12- nucleic acid base sequence of GTT GGT GTG TTG). Primer premier 5.0 (PRIMIER Biosoft International, Palo Alto, CA, USA) was used to design qRT-PCR specific amplification primers for the target genes (Seeing Table. S2).

Table S2 qRT-PCR primer parameters

6-base nucleic acid base sequence GTG TTG
F: GTGTTG
R: CAACAC
The 12- nucleic acid base sequence of GTT GGT GTG TTG
F: GTTGGTGTGTTG
R: CAACACACCAAC

All primers were synthesised by Sangon Biotech (Shanghai) Co., Ltd, and the dried primers were diluted to 10 μM/L using sterilised water and stored at 4°C or 20°C.

(2) Establishment of standard curve for qRT-PCR assay: The PCR amplification system was formulated using the primers in Table. S2, the amplification system is shown in Table S3, and the PCR reaction parameters were (95°C, 3min) + (95°C, 30s + 57°C, 30s + 72°C, 30s) × 30 + (72°C, 7min).

Table S3 Normal PCR system for target genes

systematic composition	dosage
upstream primers	1.0 μL
downstream primers	1.0 μL
2×LA Taq Mix	25 μL
DNA	2.0 μL
Sterilised water addition volume	50 μL

The amplification and melting curves of the qRT-PCR method are shown in Fig. S2

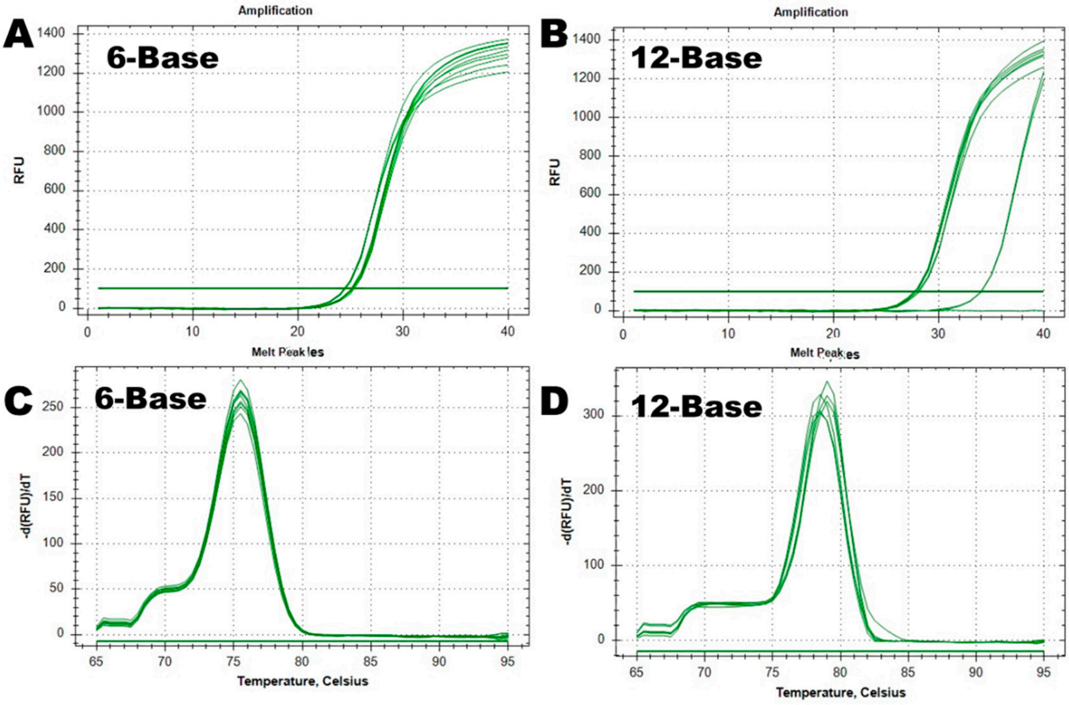

Fig. S2 (A) Amplification curve of the 6-Base; (B) Amplification curve of the 12-Base; (C) melting curve of the 6-Base; (D) melting curve of the 12-Base.

1.3 *Calibration fit curve for DNA test results*

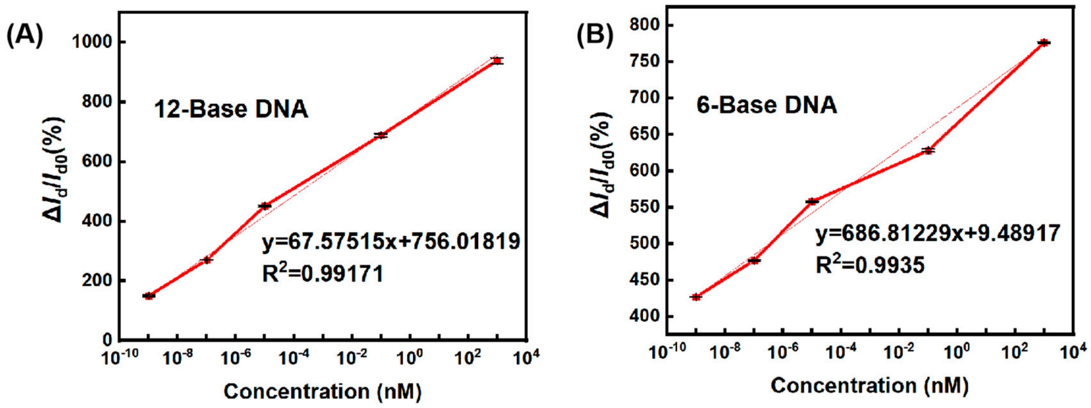

Fig. S3 (A) Calibration fit curve of the 6-Base; (B) Calibration fit curve of the 12-Base.
